# Effect of insulin resistance on CAC scores in cancer survivors

**DOI:** 10.1186/s40959-023-00168-z

**Published:** 2023-04-14

**Authors:** N. Jacobi, S. Ortman, L. Buda, Daniel Duprez

**Affiliations:** 1 Department of Hematology, Oncology, Hennepin Healthcare, Minneapolis, MN USA; 2 Department of Medicine, Hennepin Healthcare, Minneapolis, MN USA; 3 Department of Cardiology, University of Minnesota, Minneapolis, MN USA

**Keywords:** Insulin resistance, Hyperinsulinemia, CAC scans, Cancer survivors, Coronary artery disease, Coronary heart disease

## Abstract

**Background:**

Many ca. survivors exhibit signs of IR, an important risk factor for the development of CAD. CAC scans offer a risk assessment of CV disease before cardiac damage has occurred. We investigated how IR affects CAC scores in cancer survivors.

**Objectives:**

The aim of this study was to show that CAC scores differ significantly between insulin-sensitive- and -resistant cancer survivors.

**Methods:**

We enrolled 90 cancer survivors of a large community hospital from March 2021 to January 2022 into this pilot study. Patients were subdivided into three groups: insulin-sensitive (IS), insulin-resistant/prediabetic and insulin-resistant/diabetic. All patients received a CAC scan.

**Results:**

70% of asymptomatic survivors overall and 81% of asymptomatic IR patients show CAD on CAC scans. 17 CAC scans in the IS group, 6 CAC scans in the IR/prediabetic group and 5 CAC scans in the IR/diabetic group showed an Agatston score of 0. The p-value between the three groups was statistically significant (*p* = 0.005) whereas the IR/prediabetic- and the IR/diabetic group did not differ statistically from each other. The mean MESA 10-year CHD risk with CAC was 7.8. There was a highly significant difference between the 3 groups (*p* < 0.001). The two IR groups did not differ statistically (p = 0.076).

**Conclusions:**

Survivors with IR including prediabetes have less frequent zero CAC scores than insulin-sensitive survivors. Our study also showed that IR including prediabetes significantly increases the MESA 10-yr. CHD Risk with CAC in cancer survivors. This trial highlights the importance of screening survivors for IR and draws attention to the association of IR to CAC not only in diabetes but also in prediabetes. The high fraction of asymptomatic survivors with CAD is concerning and calls for further investigation. CAC scans are an inexpensive and efficient way of screening asymptomatic cancer survivors for CAD.

## Background

Hyperinsulinemia/insulin resistance (IR) in general, not only diabetes, is an important risk factor for the development of coronary artery disease (CAD) [1–5]. An important goal is to detect CAD before events such as angina, myocardial infarction (MI) and cardiac interventions have taken place. CAC scans offer an assessment of CAD in the asymptomatic patient before cardiac damage has occurred. High CAC scores increase the risk for cardiac events within the next 10 years. Framingham data has shown that 50% of coronary events are not predicted from traditional cardiac risk factors [6]. CAC scores are highly predictive for future cardiac events in asymptomatic patients according to their risk category (low, intermediate and high) [7–10]. Thus, CAC scores offer valuable information regarding a patient’s risk of developing symptomatic CAD in addition to known cardiac risk factors.

Cancer survivors are typically monitored for long-term effects caused by their treatment. A major focus is on the risk of cardiomyopathy from chemotherapy and/or radiation [11]. However, CAD can also develop due to shared risk factors or as a consequence of cancer treatment (e.g. radiation). Cancer survivors are at particular risk for cardiovascular events. Their prevalence of CAC is significantly higher than in patients without cancer [12]. It has to be suspected that this is in part due to the high incidence of IR in this population.IR is not only a risk factor for CAD but also for certain types of cancers. For example, breast cancer risk is not only increased in patients with DM but also in patients who are still in the prediabetic phase [13]. Marked IR increases the incidence of breast cancer and all-cause mortality after breast cancer in postmenopausal women in the Women’s Health Initiative [14]. Hyperinsulinemia and IR are associated with an increased risk of lung cancer [15]. Similar findings have been discovered in prostate-, colon- and pancreatic cancer [16–19]. Insulin leads to proliferation and reduction of apoptosis in colorectal cancer, shown in cell lines and animal models [20–22].

We set out to investigate the association of IR with CAC scores in cancer survivors.

## Methods

### Patients

Patients were recruited from the survivorship clinic of Hennepin Healthcare. Inclusion criteria was a previous history of cancer. Exclusion criteria were a history of CAD including angina, MI, percutaneous transluminal coronary angioplasty (PTCA) with and without stent placement, coronary artery bypass graft (CABG) and pregnancy.

### Trial design

All patients were tested for fasting insulin, fasting glucose, HgbA1c and had a lipid panel. Only those patients whose laboratory results did not indicate diabetes or prediabetes underwent an oral glucose tolerance test (oGTT).

Data were collected on demographics with age, gender, race, height, weight and BMI. Cardiac risk factors were determined (current and previous nicotine use, family history of heart attacks, current and previous history of diabetes and prediabetes). The patients’ systolic and diastolic blood pressure at the time of recruitment was documented. We calculated the Framingham cardiovascular 10-year risk score and the ACC/AHA clinical atherosclerotic cardiovascular disease (ACSVD) 10-year risk score for every patient. Additionally, we collected information on any other history of heart disease besides CAD.

We documented whether patients were on exogenous insulin. Data were obtained on lipid lowering drugs and antihypertensive use.

We obtained data on tumor characteristics such as histology, stage and recurrence. We put special emphasis on documenting cardiotoxic treatments such as chemotherapy (e.g., anthracyclines) and radiation to the left chest or both. We also gathered data on the use of aromatase inhibitors, androgen biosynthesis inhibitors and tyrosine kinase inhibitors.

### Trial conduction

Patients were recruited from March 2021 until January 2022. They were assessed according to our inclusion- and exclusion criteria. After obtaining informed consent, study participants underwent baseline laboratory tests. They were subdivided into one of three groups according to their oGTT- and HgbA1c results (insulin-sensitive, insulin-resistant/prediabetes and insulin-resistant/diabetes). All patients underwent a CAC scan shortly after baseline laboratory results were completed.

### Statistical analysis

Clinical and demographic measures were summarized descriptively and compared between the three groups using one-way *ANOVA* for continuous, non-CAC/MESA related measures, and Fisher’s Exact test for categorical measures. Due to the skewness among the CAC and MESA scores, the Kruskal-Wallis test was used for these measures. A subgroup comparison was made between the IR-prediabetic and IR diabetic groups using *t*-tests, Mann-Whitney U, or Fisher’s Exact tests, as appropriate. P-values less than 0.05 were considered statistically significant. R (R Core Team) Version 4.0 was used for all analyses.

## Results

We enrolled 90 patients into our study. 32 patients were insulin-sensitive, 29 patients were insulin-resistant/prediabetic and 29 patients were insulin-resistant/diabetic. Mean age of all trial participants was 64.1 and did not differ significantly among the three groups. 57.8% of patients were female, 42.2% were male. There was no significant gender difference between the three groups. Mean BMI was 29.1 across all study participants but differed significantly across the three groups. Mean BMI in the IS group was 26.5, in the IR/prediabetic group 28.4 and in the diabetic group 32.8 which was statistically significant (p = 0.002). The distribution of race differed significantly across the three groups (Table 1).

Mean weight was 79.6 kg across all study participants, and the difference among the three groups was statistically different (74.2 kg in IS group, 77.1 kg in IR/prediabetic group and 88.1 kg in IR/diabetic group, p = 0.006). The three groups were similar in smoking status, pack years, family history of heart attack, diagnosis of hyperlipidemia and being on antihypertensives (Table 1). The study participants had similar systolic and diastolic blood pressure levels (Table 1).

There was a statistical difference among the Framingham CV Risk for men (overall mean 23.3 (SD 13.3), *p* = 0.38). Women also showed statistically different values with an overall mean of 10.6 (8.0), *p* < 0.001) (Table 1). The ACC/AHA risk score showed an overall mean of 15 (12.3). The three groups were statistically different (IS 8.5, IR/prediabetic 14.1, IR/diabetic 23, *p* < 0.001). The three groups were comparable regarding previous cardiac history (Table 2).

**Table 1:** Patient Characteristics and Cardiac Risk Factors

	Insulin Sensitive (N = 32)	IR - Prediabetic (N = 29)	IR - Diabetic (N = 29)	Total (N = 90)	P-value
Age					0.071
Mean (SD)	60.9 (10.5)	66.1 (9.8)	65.5 (8.2)	64.1 (9.7)	
Range	45.0–87.0	43.0–82.0	49.0–82.0	43.0–87.0	
Gender - Male	11 (34.4%)	15 (51.7%)	12 (41.4%)	38 (42.2%)	0.395
Race					< 0.001
African/AA	8 (25.0%)	4 (13.8%)	13 (44.8%)	25 (27.8%)	
Asian	1 (3.1%)	1 (3.4%)	1 (3.4%)	3 (3.3%)	
Caucasian	23 (71.9%)	18 (62.1%)	8 (27.6%)	49 (54.4%)	
Hispanic	0 (0.0%)	6 (20.7%)	7 (24.1%)	13 (14.4%)	
Height (m)					0.284
Mean (SD)	1.7 (0.1)	1.6 (0.1)	1.6 (0.1)	1.7 (0.1)	
Range	1.5–1.9	1.5–1.8	1.4–1.9	1.4–1.9	
Weight (kg)					0.006
Mean (SD)	74.2 (13.1)	77.1 (16.4)	88.1 (21.6)	79.6 (18.1)	
Range	52.0–103.4	50.3–131.0	59.4–136.8	50.3–136.8	
BMI					< 0.001
Mean (SD)	26.5 (5.2)	28.4 (5.7)	32.8 (7.7)	29.1 (6.7)	
Range	19.6–39.7	18.9–48.1	22.1–50.6	18.9–50.6	
BMI (categorical)					0.029
Normal	14 (43.8%)	7 (24.1%)	4 (13.8%)	25 (27.8%)	
Overweight	12 (37.5%)	11 (37.9%)	9 (31.0%)	32 (35.6%)	
Obese	6 (18.8%)	11 (37.9%)	16 (55.2%)	33 (36.7%)	
Smoking Status					0.809
Missing	1	3	0	4	
Current	5 (16.1%)	4 (15.4%)	2 (6.9%)	11 (12.8%)	
Former	9 (29.0%)	9 (34.6%)	11 (37.9%)	29 (33.7%)	
Never	17 (54.8%)	13 (50.0%)	16 (55.2%)	46 (53.5%)	
Pack Years					0.362
Mean (SD)	23.4 (17.1)	42.0 (56.5)	22.0 (36.0)	29.0 (39.5)	
Range	0.5–70.0	0.4–200.0	1.0–130.0	0.4–200.0	
Hyperlipidemia	22 (68.8%)	21 (72.4%)	22 (75.9%)	65 (72.2%)	0.802
Family Hx Heart Attack	10 (31.2%)	4 (13.8%)	10 (34.5%)	24 (26.7%)	0.170
Total Cholesterol					0.117
Mean (SD)	196.3 (42.8)	186.0 (45.5)	172.4 (45.4)	185.3 (45.1)	
Range	99.0–299.0	91.0–281.0	88.0–277.0	88.0–299.0	
HDL					< 0.001
Mean (SD)	69.5 (19.6)	54.6 (13.3)	51.1 (13.9)	58.8 (17.8)	
Range	42.0–115.0	27.0–76.0	31.0–81.0	27.0–115.0	
Systolic BP					0.687
Mean (SD)	126.8 (15.5)	130.1 (16.4)	129.9 (18.9)	128.9 (16.8)	
Range	94.0–164.0	96.0–162.0	98.0–160.0	94.0–164.0	
Diastolic BP					0.256
Mean (SD)	75.8 (11.3)	72.1 (12.5)	71.2 (10.1)	73.1 (11.4)	
Range	52.0–100.0	44.0–98.0	50.0–93.0	44.0–100.0	
On Lipid Lowering Drugs	5 (15.6%)	7 (24.1%)	19 (65.5%)	31 (34.4%)	< 0.001
Framingham CV Risk (Men)					0.038
Mean (SD)	16.9 (8.5)	20.8 (10.0)	30.0 (15.9)	23.3 (13.3)	
Range	4.4–27.8	10.5–46.9	10.6–61.0	4.4–61.0	
Framingham CV Risk (Women)					< 0.001
Mean (SD)	7.1 (4.1)	9.1 (3.9)	17.5 (11.4)	10.6 (8.0)	
Range	2.5–17.9	2.3–16.7	4.5–47.2	2.3–47.2	
ACC/AHA (ASCVD)					< 0.001
Mean (SD)	8.5 (8.3)	14.1 (10.0)	23.0 (13.7)	15.0 (12.3)	
Range	0.8–36.3	1.2–35.1	3.8–61.6	0.8–61.6	

P-values come from ANOVA for continuous measures, and Fisher’s Exact Test for categorical measures

**Table 2:** Cancer treatment

	Insulin Sensitive (N = 32)	IR - Prediabetic (N = 29)	IR - Diabetic (N = 29)	Total (N = 90)	P-value
Cardiotoxic Treatment	13 (40.6%)	5 (17.2%)	7 (24.1%)	25 (27.8%)	0.120
Radiation L chest	13 (40.6%)	4 (13.8%)	4 (13.8%)	21 (23.3%)	0.020
Cardiotoxic Chemo	9 (28.1%)	1 (3.4%)	4 (13.8%)	14 (15.6%)	0.027
Cardiotoxic Chemo + Radiation	7 (21.9%)	0 (0.0%)	1 (3.4%)	8 (8.9%)	0.005
Cardiac Damage from Chemo	1 (3.1%)	0 (0.0%)	0 (0.0%)	1 (1.1%)	0.999
P-values come from Fisher’s Exact test					

The patients did not differ in their total cholesterol- and LDL levels. However, there was a significant difference between the three groups regarding HDL (69.5 IS, 54.6 IR/prediabetic, 51.1 IR/diabetic, *p* < 0.001). There was a difference between patients in terms of being on lipid lowering drugs (IS 15.6%, IR/prediabetic 24. % and IR/diabetic 65. %, *p* < 0.001).

Mean fasting insulin across all study participants was 23 U/ml (35.4) in all 3 groups. As expected, there was a statistical difference between the IS group (8.8), the IR/prediabetic group (22.4) and the IR/diabetic group (39.3, p < 0.003). Similar findings were observed with fasting glucose where the mean was 114.5 mg/dl (40.4) in all three groups. The IS group had a mean fasting glucose of 94.4 mg/dl, the IR/prediabetic group had a mean of 112.1, and the IR/diabetic group had a mean of 139.2, p < 0.001. We also calculated the HOMA-IR (Homeostatic Model Assessment for Insulin Resistance) by measuring fasting insulin- and glucose. The HOMA-IR formula determines the degree of IR. A level above 2 signifies IR. The overall mean of HOMA-IR was overall 5.8 (8.9), the mean in the IS group was 2.1, the mean in the IR/prediabetic group was 6.7 and the mean in the IR/diabetic group was 9.1 (*p* = 0.006). Along the same lines, HgbA1c had a mean of 6.1% (1.5) with 5.2% in the IS group, 5.7% in the IR/prediabetic group and 7.5% in the IR/diabetic group (*p* < 0.001). The oral glucose tolerance test had a mean of 94.7 in the IS group and 162.7 in the IR/prediabetic group (Table 3).

**Table 3:** Lab results

	Insulin Sensitive (N = 32)	IR - Prediabetic (N = 29)	IR - Diabetic (N = 29)	Total (N = 90)	P-value
Cholesterol					0.117
Mean (SD)	196.3 (42.8)	186.0 (45.5)	172.4 (45.4)	185.3 (45.1)	
Range	99.0–299.0	91.0–281.0	88.0–277.0	88.0–299.0	
HDL					< 0.001
Mean (SD)	69.5 (19.6)	54.6 (13.3)	51.1 (13.9)	58.8 (17.8)	
Range	42.0–115.0	27.0–76.0	31.0–81.0	27.0–115.0	
LDL					0.287
Mean (SD)	107.1 (37.4)	105.5 (41.2)	92.5 (37.7)	101.9 (38.9)	
Range	41.0–195.0	30.0–191.0	6.0–163.0	6.0–195.0	
Exogenous Insulin	0 (0.0%)	0 (0.0%)	17 (58.6%)	17 (18.9%)	< 0.001
Fasting Insulin					0.003
Mean (SD)	8.8 (4.3)	22.4 (31.3)	39.3 (49.7)	23.0 (35.4)	
Range	2.6–21.6	2.6–168.0	3.0–172.0	2.6–172.0	
Fasting Glucose					< 0.001
Mean (SD)	94.4 (7.3)	112.1 (16.3)	139.2 (61.3)	114.5 (40.4)	
Range	79.0–106.0	73.0–141.0	6.9–291.0	6.9–291.0	
HOMA-IR					0.006
Mean (SD)	2.1 (1.1)	6.7 (10.9)	9.1 (10.2)	5.8 (8.9)	
Range	0.6–5.0	0.8–58.5	0.8–48.1	0.6–58.5	
HgbA1c					< 0.001
Mean (SD)	5.2 (0.2)	5.7 (0.4)	7.5 (1.9)	6.1 (1.5)	
Range	4.4–5.6	4.8–6.2	5.2–12.6	4.4–12.6	
oGTT					
N	32	11	0	43	
Mean (SD)	94.7 (28.0)	162.7 (25.1)	-	112.1 (40.4)	
Range	47.0–138.0	125.0–199.0	-	47.0–199.0	

P-values come from ANOVA for continuous measures, and Fisher’s Exact Test for categorical measures

Mean baseline CAC among all three groups was 169.5 (359.8). The overall median was 27.5 (0.0, 2316.0). The IS group had a mean of 202.4 (520.6), the IR/prediabetic group showed a mean of 183.9 (257.5), and the IR/diabetic group had a mean of 119.0 (202.4), *p* = 0.029. The medians of the three groups were 0.0 (0.0, 2316.0), 74.0 (0.0, 977.0) and 39.0 (0.0, 969.0), respectively. There was no statistical difference between the IR/prediabetic- and the IR/-diabetic group (*p* = 0.404).

**Fig. 1:**
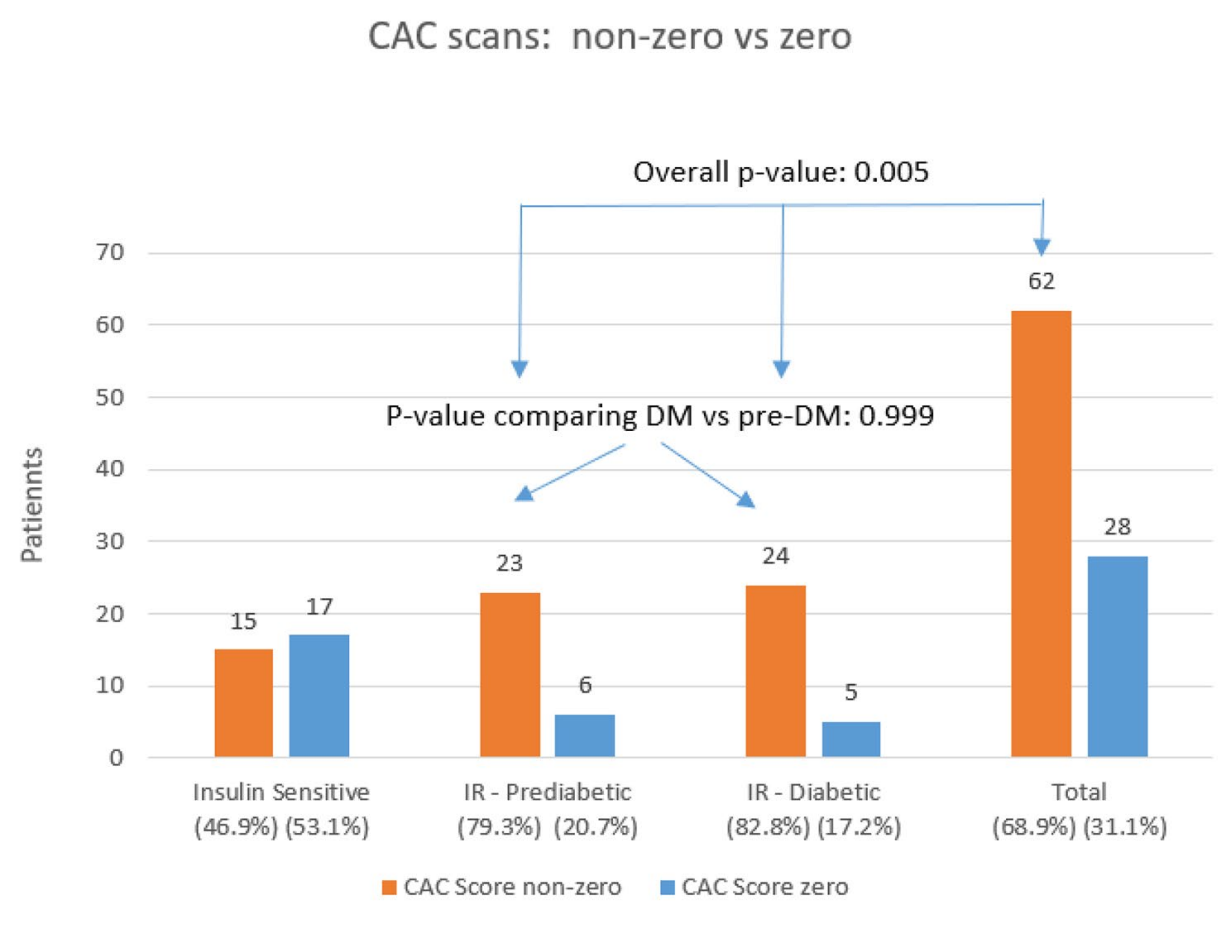
Zero- versus non-zero CAC scans in the IS- and IR groups

70% of asymptomatic survivors overall and 81% of asymptomatic IR patients show CAD on CAC scans. The IS group showed 17 zero (53.1%) and 15 (46.9%) non-zero CAC scan values, the IR/prediabetic had 6 zero (20.7%) scans and 23 (79.3%) non-zero scans, and the IR/diabetic group contained 5 (17.2%) patients with zero scans whereas 24 (82.8%) CAC scans were non-zero, *p* = 0.005. The IR/prediabetic- and the IR/diabetic group did not differ statistically from each other (*p* = 0.999). The difference in non-zero CAC score values among all 3 groups was not statistically significant (*p* = 0.388) (Fig. 1).

**Table 4:** CAC results

	Insulin Sensitive (N = 32)	IR - Prediabetic (N = 29)	IR - Diabetic (N = 29)	Total (N = 90)	Overall P-value	P-value Comparing DM vs. pre-DM
CAC-Agatson Score					0.029^a^	0.404^a^
Mean (SD)	202.4 (520.6)	183.9 (257.5)	119.0 (202.4)	169.5 (359.8)		
Median (Range)	0.0 (0.0, 2316.0)	74.0 (0.0, 977.0)	39.0 (0.0, 969.0)	27.5 (0.0, 2316.0)		
CAC-Agatson Score*					0.005^a^	
Mean (SD)	81.6 (205.2)	183.9 (257.5)	119.0 (202.4)	127.6 (224.4)		
Median (Range)	0.0 (0.0, 876.0)	74.0 (0.0, 977.0)	39.0 (0.0, 969.0)	23 (0.0, 977)		
CAC-Agatson Score (non-zero)					0.388^a^	0.148^a^
N	15	23	24	62		
Mean (SD)	431.7 (702.9)	231.8 (269.7)	143.8 (214.9)	246.1 (412.0)		
Median (Range)	79.4 (2.0, 2316.0)	158.0 (0.9, 977.0)	69.5 (1.0, 969.0)	94.5 (0.9, 2316.0)		
CAC-Agatson Score (non-zero)*					0.321^a^	
N	13	23	24	60		
Mean (SD)	188.3 (282.8)	231.8 (269.7)	143.8 (214.9)	187.2 (250.9)		
Median (Range)	64.0 (2.0, 876.0)	158.0 (0.9, 977.0)	69.5 (1.0, 969.0)	86.2 (0.9, 977.0)		
CAC Score (binary)					0.005	0.999
Non-zero	15 (46.9%)	23 (79.3%)	24 (82.8%)	62 (68.9%)		
Zero	17 (53.1%)	6 (20.7%)	5 (17.2%)	28 (31.1%)		
Mesa baseline %ile					0.041	0.958
Mean (SD)	36.2 (41.2)	56.3 (32.9)	56.8 (31.9)	49.3 (36.7)		
Median (Range)	0.0 (0.0, 99.0)	64.0 (0.0, 93.0)	59.0 (0.0, 98.0)	59.5 (0.0, 99.0)		
Mesa 10-Year CHD risk w/ CAC					< 0.001	0.045
Mean (SD)	5.3 (6.6)	7.3 (5.1)	11.0 (8.3)	7.8 (7.1)		
Median (Range)	2.1 (0.9, 27.6)	5.5 (1.0, 18.8)	6.6 (1.5, 35.1)	5.5 (0.9, 35.1)		
Mesa 10-Year CHD risk w/o CAC					< 0.001	< 0.001
Mean (SD)	4.2 (3.6)	5.7 (3.5)	10.9 (6.5)	6.8 (5.5)		
Median (Range)	2.4 (0.7, 14.4)	4.9 (0.9, 15.4)	9.0 (2.3, 24.6)	5.3 (0.7, 24.6)		

P-values come from ANOVA for continuous measures, and Fisher’s Exact Test for categorical measures

The total mean MESA baseline percentile was 49.3%. The IS group had a mean of 36.2%, the IR/prediabetic group a mean of 56.3% and the IR/diabetic group a mean of 58.8% which was not statistically different (p = 0.168).

The mean MESA 10-year CHD risk with CAC was 7.8. There was a highly significant difference between the 3 groups: the IS group had a mean of 5.3, the IR/prediabetic group had a mean of 7.3, and the IR/diabetic group had a mean of 11.0, p < 0.001. The two IR groups did not differ statistically (p = 0.076) (Table 4).

## Discussion

Our study showed that IR including prediabetes reduces a cancer survivor’s probability of having a zero CAC scan. The statistical difference between the two IR groups in regard to the probability of having a non-zero CAC scan was not significant in our trial. IR including prediabetes also significantly increased the MESA 10-yr risk with CAC. There was no statistical difference between the two IR groups. Other authors have shown that IR increases CAC scores in a normal patient population. Similar to our small trial on cancer survivors, an increase in number of non-zero scans in association with HOMA-IR was observed in large number of non-diabetic patients in the MESA trial. Like in our study there was no association with extent of calcification [23]. This was in contrast to patients in the Framingham Offspring study where extent of subclinical CAD was associated with severity of IR [24].

The focus of our trial was on the population of cancer survivors. Cancer survivors disproportionately exhibit IR. One possible reason is the association of certain types of cancer in patients with hyperinsulinemia [13–19].

The similarities between the two IR groups in terms of the risk of having a non-zero CAC scan and the increased risk of the MESA 10-year CHD with CAC raise the question about the importance of screening survivors for IR including prediabetes. The strength of our study lies in the meticulous distinction between the two insulin-resistant groups through various laboratory tests. It should be kept in mind that IR can be missed on conventional laboratory tests such as fasting glucose and HgbA1c. Given the significant number of patients diagnosed with prediabetes based solely on oGTT in this trial, future effort should be made to evaluate the benefit of screening cancer survivors with oGTT.

Almost 70% of our asymptomatic study participants showed CAD on CAC screening. Moreover, the fraction of asymptomatic IR patients is even higher with 81%. These numbers are certainly concerning and call for further investigation on a larger scale. In view of the increased cardiovascular risk of cancer survivors, some authors have suggested prospective trials on screening survivors with cardiac tomographies (including CAC scans and coronary CT angiography) for surveillance [25]. There is a need for screening cancer survivors for CAD above that of the general population. Yet, monitoring cancer survivors with coronary CT angiographies could lead to financial toxicity for the patients. CAC scans are a less expensive and efficient way of screening cancer asymptomatic survivors for CAD as shown in this pilot trial.

The CAC consortium has demonstrated that high CAC scores preceding a cancer diagnosis was predictive of having CVD as a supporting cause of death on death certificates. The risk through elevated CAC scores was independent of the Framingham ASCVD sore and general CVD risk factors. The authors also encouraged the utilization of CAC scans as a screening tool for cancer survivors [26]. This is especially important for patients with high CAC scores as it has been shown that in patients with CAC scores > 300 death from CAD even surpasses death from cancer [27].

The association between CAC scores and certain types of cancer is notable. A cancer diagnosis is associated with the development of CAC independent of other cardiac risk factors in patients with a zero CAC at baseline [28]. Very high CAC scores are also associated with high incidences of cancer [29]. In addition, CAC scores are associated with an increased risk for lung cancer mortality, especially in current/former smokers and women [30].

Moreover, CAC scans are prognostic of major adverse cardiac events and all-cause deaths in patients with lung cancer without a history of CVD [31]. CAC scores were found to be an independent predictor of CV events and all-cause mortality on low-dose lung CT’s [32]. Similar observations were made in breast cancer patients after adjuvant radiation therapy where high pre-treatment CAC scores were associated with acute coronary events [33].

Many chemotherapeutic agents and left-sided chest irradiation are recognized as cardiotoxic. Yet, left-sided chest radiation has not been shown to increase CAC scores in breast cancer survivors [34].

Further studies are needed to evaluate whether we are overestimating the effect of cardiotoxic treatment and/or radiation and underestimating the effect of IR on CAD in cancer survivors.

## Conclusions

In our trial, asymptomatic cancer survivors show a high rate of CAD on CAC scans. IR including prediabetes and not only diabetes increased the risk of a non-zero CAC scan in cancer survivors. IR including prediabetes also significantly increased the 10-year MESA CHD risk with CAC. Prediabetes appears to be a more significant cardiac risk factor for survivors than anticipated. Further trials should investigate whether cancer survivors need to be tested for IR with yearly oGTT’s and furthermore, whether asymptomatic survivors with IR should undergo screening CAC scans.

### Study limitations

This is a single-center pilot study of a specific population of cancer survivors at a safe-haven hospital. Further studies are required to confirm that these data can be extrapolated to all cancer survivors. Moreover, multivariate analyses are called for to validate IR as an independent risk factor. Research is also needed as to the rate of progression of CAC scores in cancer survivors in association with IR.

## Data Availability

The datasets used and/or analysed during the current study are available from the corresponding author on reasonable request.

## Abbreviations

ca.: cancer
IR: insulin resistance
CAD: coronary artery disease
CAC: coronary artery calcium
CV: cardiovascular
IS: insulin-sensitive
oGTT: oral glucose tolerance test
MESA: Multi-Ethnic Study of Atherosclerosis
MI: myocardial infarction
HR: hazard ratio
PTCA: percutaneous transluminal coronary angioplasty
CABG: coronary artery bypass graft
ACSVD: atherosclerotic cardiovascular disease
ACC/AHA: American College of Cardiology/American Heart Association
BMI: body mass index
HgbA1c: hemoglobin A1C
CT: computed tomography
